# Takayasu Arteritis: What Can Go Wrong in The Glomeruli for Large Vessel Vasculitis? A Case Report of an Unusual Cause of Persistent Microscopic Hematuria in a Patient with Takayasu Arteritis

**DOI:** 10.7759/cureus.5024

**Published:** 2019-06-28

**Authors:** Boon Cheok Lai, QinHao Jonathan Ye, Tin Kyaw Kyaw Aung

**Affiliations:** 1 Internal Medicine, Sengkang General Hospital, Singapore, SGP

**Keywords:** takayasu arteritis, glomerulonephritis, microscopic hematuria

## Abstract

We describe a case of persistent microscopic hematuria in a patient with Takayasu arteritis (TA). Urological cause has been excluded. Classically, TA is found to only involve large arteries like the aorta and its branches. There is some evidence that showed the association of small vessel vasculitis like glomerulonephritis with TA. Histopathological studies showed similar features of vasculitis between the small and large vessel involvement in TA. This may explain the similar disease process that has not been well understood in TA. We believe that the persistent microscopic haematuria in the patient described is caused by TA associated glomerulonephritis. A series of investigations ruled out other causes of glomerular microscopic haematuria like autoimmune or infection related glomerulonephritis.

## Introduction

Takayasu arteritis (TA) is a rare, chronic systemic vasculitis of unknown etiology that primarily affects the aorta and its primary branches [1]. Based on the International Chapel Hill Consensus Conference (CHCC) classification, TA rarely affects small vessels like the glomerulus that would potentially lead to the presentation of microscopic hematuria [2]. However, there have been some case reports about the association of TA with glomerulonephritis.

## Case presentation

A 63-years old Chinese woman was referred to the Renal Medicine clinic for persistent microscopic hematuria by the urology service. She had a past medical history of hypertension for three years and her blood pressure (BP) was well controlled on telmisartan and nifedipine. Prior to the review, she had been worked up by urology with a computed tomography (CT) urogram that did not show any renal calculi or tumor which could account for the hematuria.

Her serum creatinine was 53 umol/L, 24 hours total urine protein 0.09 g/day; microscopic examination of the urine showed red blood cells (RBCs) 23/UL, white blood cells 0/UL and epithelial cells 0/UL. Urine phase contrast studies showed 80% of dysmorphic and 20% of isomorphic red blood cells. Other relevant work up for her microscopic hematuria was normal. Her serum complement 3 and complement 4 were normal. Serum antinuclear antibody, anti-double-stranded deoxyribonucleic acid (DNA) antibody and anti-neutrophil cytoplasmic antibody were negative. Anti-human immunodeficiency virus (HIV) antibody, hepatitis B surface antigen, and anti-hepatitis C virus antibody were negative as well.

**Figure 1:**
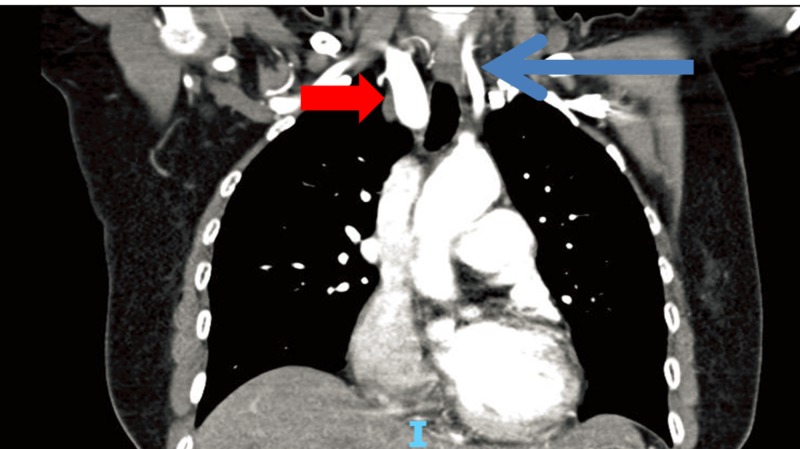
Computed tomography (CT) showing aneurysmal dilatation of the right subclavian artery (red arrow) as compared to the left subclavian artery (blue arrow)

**Figure 2:**
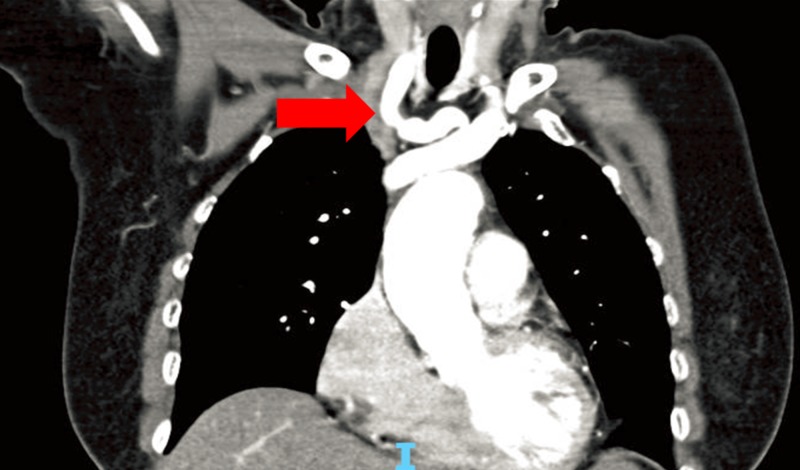
Red arrow showing fusiform dilatation of the right subclavian artery

**Figure 3:**
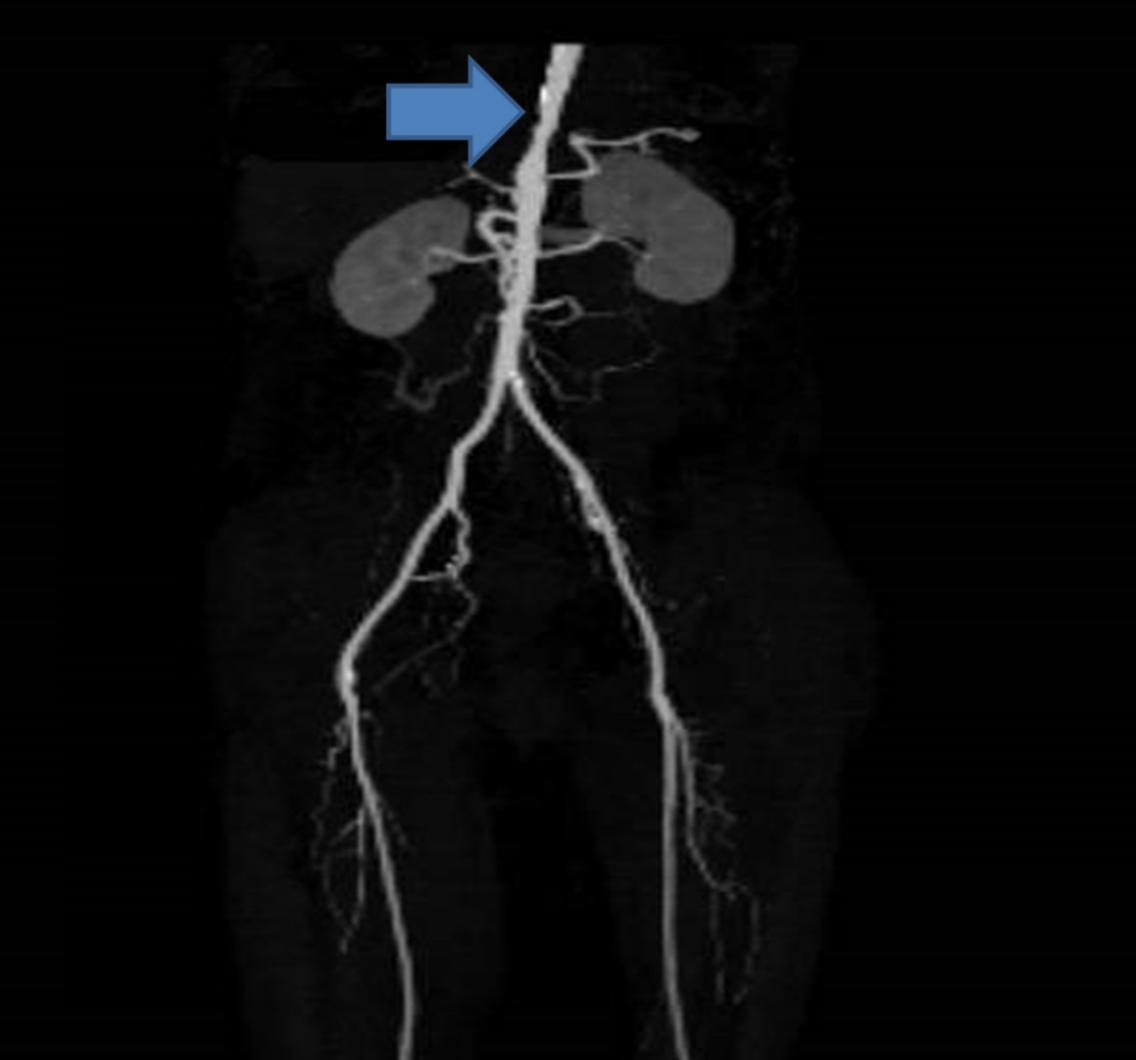
Blue arrow showing focal stenosis of the descending aorta just above the aortic hiatus

Incidentally, she was found to have different BP readings in her arms during the clinic visit. Her left arm BP was 170/70 mmHg and right arm BP 130/56 mmHg. Physical examination showed reduced pulse volume over the right upper limb as compared to the left upper limb. Pulse volume in both the lower limbs was equal. An urgent CT angiogram was performed to rule out coarctation of the aorta and it showed multiple stenoses and aneurysmal changes of the aorta and its main branches, suggestive of TA. An angiogram was carried out and showed penetrating ulcers in the distal descending thoracic and infrarenal abdominal aorta. (Figures 1-3)

Further history revealed that she had a significant weight loss of 7 kg (12% of her body weight) over the past six months. She had no other symptoms associated with TA like arthralgia, carotidynia, limb claudication, cardiac angina, abdominal pain, visual impairment or dyspnea. Relevant blood test for TA disease activity including C-reactive protein (0.9 mg/L) and erythrocyte sedimentation rate (ESR) (11 mm/Hr) were normal. Other work-up for aortitis like tuberculosis quantiferon was negative.

**Figure 4:**
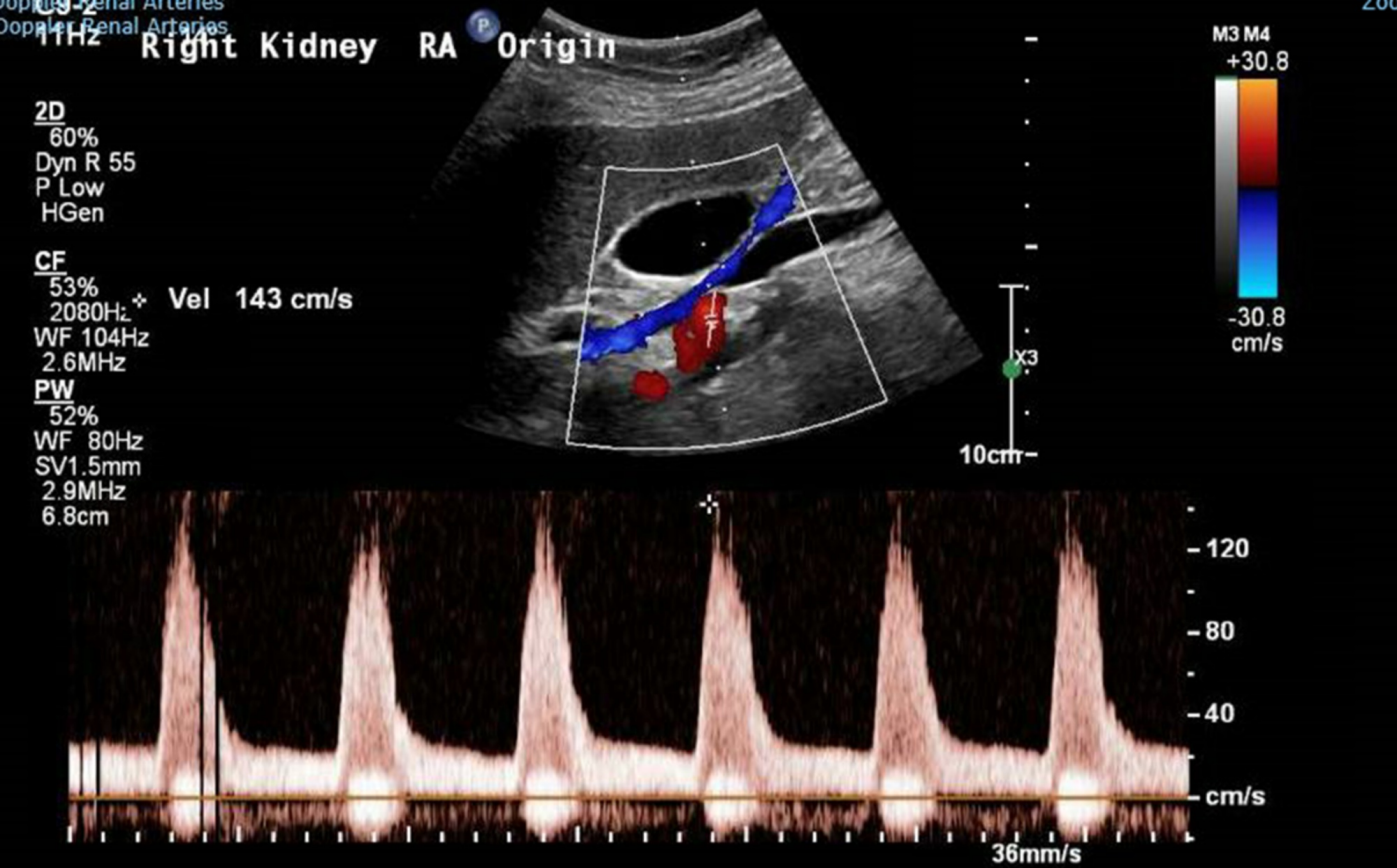
Doppler renal artery showing no intrarenal parvus tardus pattern to suggest renal artery stenosis

Further investigations were carried to evaluate the involvement of other large arteries. 2D echocardiogram showed normal left ventricular size with a normal left ventricular ejection fraction. It did not show any evidence to suggest ascending aortitis. Carotid Doppler ultrasound showed 25%-30% stenosis of both carotid arteries but doppler ultrasound of the renal arteries showed no evidence of hemodynamically significant stenosis (Figure 4). She had fluorodeoxyglucose (FDG) positron emission tomography (PET)/CT scan done and showed no abnormal metabolic activity, particularly in the arterial wall to suggest arteritis.

In view of the lack of signs of active inflammation and systemic symptoms associated with TA, the decision was made for conservative management and immunosuppression was not given. Follow-up consultations over the past year did not show any further weight loss and the patient remains symptom free. Repeat CT angiogram did not show progression of her TA as well. Despite the persistent microscopic hematuria, her kidney function remained normal and proteinuria remained less than 0.3 g/day. Renal biopsy was not performed as her renal condition remains stable and management would remain supportive and not for immunosuppressant.

## Discussion

TA is a rare systemic vasculitis that involves large-size arteries with a worldwide incidence of about 2.6 cases per million population per year [1]. This disease can be seen worldwide but the majority of patients with this disease are found in Asia [3-4], especially in Japan which has an incidence of 100-200 cases diagnosed every year [5]. Studies have revealed that this increased incidence in Japan could be explained by the presence of HLA Bw52 gene in the Japanese [6]. Most cases occur in women between the age of 10 to 40 years old [7].

Our patient is female but she was diagnosed at a much later age of 63 and on further history taking, had no genetic relations to Japanese. She is also relatively asymptomatic except for her loss of weight over the past six months. The diagnosis was made incidentally when an unequal blood pressure on both arms were found during her clinic visit.

The diagnosis of TA was made as she passed ≥ 5 points of the inclusion criteria of the latest Large-Vessel Vasculitis Classification criteria from 2018 American College of Rheumatology and Association of Rheumatology Health Professionals (ACR/ARHP) [8].

- Female gender (1 point)

- Systolic blood pressure difference in arms ≥ 20mmHg (1 point)

- Affected right subclavian artery, left axillary artery, ascending aorta (total 3 points)

- Abdominal aorta and coeliac artery (3 points)

She also fulfilled 3 out of 6 criteria from the 1990 ACR classification [9].

- Decreased pulsation of one or both brachial arteries

- Difference of at least 10 mmHg in systolic blood pressure between the arms

- Arteriographic narrowing or occlusion of the entire aorta, its primary branches, or large arteries in the proximal upper or lower extremities, not due to arteriosclerosis, fibromuscular dysplasia, or other causes

Based on the CHCC classification criteria for large vessel vasculitis, TA only involves the aorta and its main branches [2]. However, several studies had shown the association of TA with glomerulonephritis, which is a small vessel vasculitis. A case series by Morales et al. showed that renal abnormalities have been found in up to 46% of TA patients [10].

Renal complications in TA may be due to vascular hypertension and ischemia, i.e., ischemic nephropathy from abdominal aorta aneurysm or stenosis of a branch of renal artery. In our patient, renal artery Doppler and CT angiogram did not show any renal artery stenosis.

There are several case reports that have found underlying biopsy-proven glomerulonephritis in patients with TA, after excluding those with ischemic renal complications.

Pablo found that 56% of TA patients had underlying glomerulonephritis from post-mortem examination (14 out of 25 patients) after excluding the ischemia renal complication arising from TA. Among all the glomerulonephritis, 71.4% (10 out of 14 patients) of them showed diffuse mesangial proliferative glomerulonephritis with vascular inflammatory cell infiltrate. This study suggests that primary glomerular lesions may not be as rare as previously reported, and the incidence of glomerulonephritis in patients with TA may be underestimated [11].

Li et al. reported six cases of patients with TA who have underlying glomerular changes as well. All of the patients had renal artery stenosis ruled out using renal artery Doppler; 50% of the patients had histology features of mesangial proliferative changes and infiltration of the inflammatory cell in the blood vessel [12].

In TA, the histological findings of the large artery show the presence of mononuclear cells infiltration into the tunica media, destruction of the elastic lamina and the muscular media that lead to aneurysmal dilatation of the affected vessel. Sometimes, progressive inflammation and dense scarring may contribute to the stenosis of the artery [13]. The histological changes in both small vessels (glomeruli) and large vessels (aorta) in TA are quite similar which may suggest a common systemic autoimmune disease that has not been well understood.

With these preceding case reports showing glomerulonephritis in patients with TA, we think our patient had glomerulonephritis as well. Despite the lack of renal biopsy, urological causes of haematuria have been ruled out and the presence of dysmorphic RBCs in the urine strongly suggest the presence of the underlying glomerulonephritis. As mentioned, we did not proceed with the biopsy as her renal function is stable and TA disease remains quiescent; the renal biopsy will not alter medical management.

## Conclusions

Despite previously understood characteristics of TA that classically only affects the large vessel like aorta and its branches, there are increasing evidence showing the involvement of the small vessels as well. In a TA patient, it is important to screen for microscopic haematuria and carry out renal biopsies if indicated to confirm the underlying glomerulonephritis. This can help us improve our understanding of the common pathology between TA (large vessel vasculitis) and glomerulonephritis (small vessel vasculitis).
